# Synthesis of Solar Light Active Reduced Graphene Oxide-ZnS Nanomaterial for Photocatalytic Degradation and Antibacterial Applications

**DOI:** 10.3390/mi14071324

**Published:** 2023-06-28

**Authors:** B. Sathya priya, Kanakaraj Aruchamy, Tae Hwan Oh, Balakrishna Avula, Imran Hasan, M. Shanthi

**Affiliations:** 1 Department of Chemistry, Annamalai University, Annamalainagar 608002, Tamil Nadu, India; 2 School of Chemical Engineering, Yeungnam University, Gyeongsan 38436, Republic of Korea; 3 Department of Chemistry, Rajeev Gandhi Memorial College of Engineering and Technology (Autonomous), Nandyal 518501, Andhra Pradesh, India; 4 Department of Chemistry, College of Science, King Saud University, Riyadh 11451, Saudi Arabia

**Keywords:** rGO-ZnS, NBB dye, degradation, solar light, antibacterial studies

## Abstract

Good water quality is essential for life; therefore, decolorizing and detoxifying organic dye wastes (textile effluents) have gained immense environmental importance in recent years. Thus, the degradation of wastewater has become a potential need for our environment. This research aims to synthesize and investigate a ceramic-based nanomaterial catalyst for the degradation of dye solution under exposure to sunlight. A reduced graphene oxide-ZnS (rGO-ZnS) nanomaterial was qualitatively synthesized using a solvothermal method. The prepared nanomaterial was characterized using XRD, SEM, HR-TEM, EDX, XPS, and FT-IR techniques. The photocatalytic activity of the rGO-ZnS nanomaterial was checked using oxidative photocatalytic degradation of naphthol blue black dye (NBB) under direct sunlight irradiation. Here, the rGO/ZnS composite showed a significant photocatalytic performance to degraded NBB (93.7%) under direct solar light. Chemical Oxygen Demand (COD) measurements confirmed the mineralization of the dye. The influence of different radical scavengers on NBB degradation was studied. Optimum conditions for efficient degradation were determined. The antibacterial property of the prepared catalyst was studied.

## 1. Introduction

Due to the rapid development of industry and the significant number of pollutants produced, including organic solvents, oils, and dyes, that have been discharged into water supplies, water pollution is now a big problem. Pollution is released into water supplies, causing a shortage of freshwater which is necessary for the survival of the world’s population [1,2]. Owing to their non-biodegradable nature, harmful effects on marine life, and mutagenic effects on humans, synthetic dyes containing aromatic compounds are extremely poisonous, difficult to degrade [3,4], and are the primary cause of environmental contamination. The search for highly active and light-source-responsive (UV/solar light) photocatalysts for efficient environmental cleanup is ongoing due to constantly expanding environmental concerns [5,6,7]. Semiconductor-intervened photocatalytic processes stand out due to their high proficiency, eco-benevolence, low speculation, simplicity of activity, and extraordinary potential for viable applications in natural remediation and maintainable energy generation [8,9,10,11,12,13]. While semiconductor nanomaterials can utilize noticeable or bright light as an illuminating source, they can prompt electron shifts to the conduction band (CB) from the valence band (VB), bringing about the formation of isolated charge transporters that can fuel different substance responses on the outer layer of semiconductor particles. Metal sulfides have recently been found to have enormous photocatalytic efficiency [14,15,16,17].

For example, zinc sulfide (ZnS), one of the primary semiconductors found, has been comprehensively examined as a photocatalyst by the scientific exploratory community [18,19]. ZnS nanomaterials have been demonstrated in experiments to be phenomenal UV-light-resolved photocatalysts because of their remarkable optical properties, including high photoexcitation charge carrier generation rates, highly negative photoexcited electron reduction potentials, and high photo-corrosion stability. There are three critical issues that should be addressed to deal with the photocatalytic execution of photocatalysts: (i) the recombination speed of photogenerated charge carriers, (ii) the fitting excitation recurrence, and (iii) the photocatalyst adsorption limit [20,21,22]. ZnS nanoparticles capped with watermelon rind extract and their potential application in dye degradation were studied by Lakshmipathy et al. [23]. To create ZnS nanomaterials with high photocatalytic activity, a range of studies based on the above-mentioned aspects have been described [24,25]. These investigations included loading with metallic nanoparticles or non-metal components and incorporating them with different semiconductors. The application of semiconductor nanoparticle loading on carbon nanomaterials for the photocatalytic destruction of organic contaminants has been extensively studied [26,27,28]. Due to its exceptional qualities, including a huge specific surface area, great thermal and electrical conductivity, superior mechanical stability, and good optical transparency, graphene, a novel type of 2D carbon nanomaterial, has attracted a lot of attention. Numerous methods for fabricating graphene and related modified graphene-based materials have been introduced since the discovery of graphene in 2004, including the exfoliation of graphite [29], chemical vapor deposition [30], epitaxial development [31], and reduction of exfoliated graphene oxide [32]. Titania-decorated reduced graphene oxide (TiO_2_x%rGO (x = 0, 1, 05, 10, 15, 20, and 25) nanocomposites (NCs) were fabricated using a hydrothermal process and characterized using various techniques [33]. Graphene oxide sheets contain numerous reactive oxygen-containing groups that are advantageous for creating graphene-based nanomaterials; therefore, one of these synthesis methods is thought to have the greatest experimental research and practical application value [34]. Photocatalytic reduction of Cr^6+^ by ZnO decorated on reduced graphene oxide (rGO) nanocomposites was reported by Srirattanapibul et al. [35]. Because of the distinct and stable features of graphene, its incorporation into nanomaterials can significantly enhance photocatalytic activity. Enhancement of photocatalytic capacity using Mn_3_O_4_ spinel ferrite decorated graphene oxide nanocomposites was reported by Imboon et al. [36]. Liu et al. demonstrated the improved photocatalytic activity of an aerogel composed of BiOBr and reduced graphene oxide (BiOBr/rGO) [37]. They revealed that the aerogel’s large surface area, spongy texture, and effective separation of photogenerated charge carriers were all responsible for the material’s enhanced photocatalytic activity.

The large surface area of graphene can be utilized as an optical lattice to trap other co-impetus nanoparticles and keep them from massing together. Graphene is believed to be the ideal electron move extension and electron sink, which could significantly speed up electron–hole pair partition and forestall recombination. It is thought to be critical to the photocatalytic cycle because it allows more photoinduced electrons to move to photocatalyst surfaces and structures; various receptive sustainable designs have participated in photocatalytic responses. Despite graphene’s large surface region, the solid electrostatic and stacking connections between graphene and natural atoms contribute to graphene’s exceptional ability to adsorb natural particles [38,39,40,41]. Gathering ZnS nanoparticles on graphene sheets is, thus, thought to be a reasonable and compelling strategy for combining elite-performance photocatalysts and is bound to be used in genuine natural remediation. Kanti das et al. reported that the rGO/Ag nanocatalyst was prepared using the green synthesis method [42].

We effectively synthesized rGO sheets with ZnS nanoparticles (rGO/ZnS) in this study. The degradation of an azo dye, naphthol blue black (NBB) dye, was used as a model water pollutant for assessing the photocatalytic activity of the produced nanoparticles. The findings show that the rGO/ZnS nanocomposite has the potential to function as an efficient and adaptable photocatalyst for the photodegradation of organic dyes in industrial wastewater.

## 2. Materials and Methods

Otto Chemie Pvt Ltd. (Mumbai, Maharashtra, India) was the supplier of graphite powder (325 mesh, 99.9%). The azo dye, NBB, was purchased from Sigma-Aldrich, India. The chemical structure of the NBB dye is given in Figure S1 (see Supplementary Materials). All of the reagents were of the analytical variety and were utilized directly after arrival without further purification. Water that has been twice distilled was utilized to make the test solution. Utilizing H_2_SO_4_ and NaOH, the pH of the solutions was changed.

### 2.1. Analytical Techniques

An X’Pert PRO diffractometer (Cu-Ka, 1.5406 A, Alemo, The Netherlands) was applied to obtain the powder X-ray diffraction (XRD) pattern. The catalyst morphological traits were examined using a JEOL-JSM-IT 200 scanning electron microscope (SEM, JEOL, Tokyo, Tokyo, Japan) equipped with OXFORD energy dispersive X-ray spectrum (EDS, Japan). The morphology and crystallinity were studied with a high-resolution Transmission Electron Microscope (HR-TEM) (the grid was dried naturally and analyzed with a JEOL-2100+ High-Resolution Transmission Microscope). Fourier Transfer Infrared (FT-IR) spectra of the samples were recorded using a spectrometer in KBr pellet holders. For X-rays, a Photoelectron Spectra (XPS), model- ESCA-3 Mark II, AlKα radiation (1486.6 eV) (VG Scientific Ltd., London, UK) was used.

### 2.2. Irradiation Experiments

Solar photocatalytic degradation studies were performed between 11 a.m. and 2 p.m. on sunny days. The reaction vessel was a 50 mL open borosilicate glass tube with dimensions of 40 cm in height by 12.6 mm in diameter. Before illumination, 50 mL of NBB (2 × 10^−4^ M) with the required amount of catalyst was agitated in the dark for 30 min. An open-air irradiation process was used to provide oxygen and ensure that the reaction solution was thoroughly mixed. There was no evidence of solvent volatility during the illumination period.

The amount of reaction mixture that was irradiated in each case was 50 mL. About 2–3 mL of the sample was taken and centrifuged at predetermined intervals to separate the catalyst. The dye degradation was monitored with absorption maxima of the dye at 320 nm, which is the characteristic absorption of the NBB dye corresponding to the aromatic part of the dye molecules, and its decrease in absorbance concerning the time of irradiation indicates the degradation of the dye. The solar intensity was 1250 × 100 ± 100 lux, measured using the LT Lutron LX-10/A digital lux meter (Taiwan). Chemical oxygen demand (COD) estimations were made with the reported procedure [43].

### 2.3. Synthesis of GO

Hummer’s process was refined to produce graphene oxide from graphite powder [44]. A total of 1 g of graphite powder and 1 g of NaNO_3_ powder were mixed in a 500 mL beaker. A pre-cooled mixture of conc. H_2_SO_4_ was poured in while continuously stirring. Then, 6 g of KMnO_4_ was gradually added in steps while the mixture was kept at 25 °C, and it was agitated constantly for 2 h to induce an exothermic reaction that caused the temperature to rise from 80 °C to 100 °C. Once the solution had reached room temperature, 300 mL of deionized water containing 50 mL of H_2_O_2_ (30%) was subsequently added. The solution was then left overnight for decantation to produce solid precipitation. To get rid of the metal ions and dust, the product was washed 5 to 7 times in a 5% HCl solution. To obtain a black powder, the solutions were centrifuged for 30 min at 4000 rpm, washed with ethanol, and filtered. Finally, a black pure graphene oxide nanosheet was produced through drying the resulting powder at 80 °C for 24 h.

### 2.4. Synthesis of rGO

GO (1.0 g) was added to create colloidal dispersion in distilled water while being constantly stirred in a magnetic stirrer at 350 °C. The colloidal solution of graphene oxide (GO) was heated and stirred for three hours after that hydrazine solution (a reducing agent) was added. A dark pasty substance of reduced graphene oxide (rGO) was obtained after filtering [45].

### 2.5. Synthesis of rGO-ZnS

rGO-ZnS nanocomposite was produced by means of dispersing 0.6 g of rGO in 100 mL of double-distilled water and ultrasonically dispersing the mixture for 60 min to create a homogenous rGO dispersion. Separately, 11.358 g of zinc nitrate hexahydrate was dissolved in 100 mL of water (0.4 M) and 3.122 g of sodium sulphide was dissolved in 100 mL of water (0.4 M), and these two solutions were combined under stirring. It was further stirred for 2 h. Zinc sulfide suspension was obtained. Then, 100 mL of the rGO suspension was added to the ZnS suspension, which was then stirred for 6 h and sonicated for 3 h. Then, the mixture was hydrothermally treated for 6 h at 180 °C in an autoclave constructed of stainless steel lined with Teflon. The precipitate (rGO-ZnS) was collected, filtered, extensively washed with ethanol, and dried at 60 °C in an air oven for 2 h. The rGO-ZnS catalyst was collected and used for further analysis. This catalyst contained 13.3 wt% of rGO. Catalysts with 9.3, 11.4, 15.2, and 17.0 wt% of rGO were prepared using the same procedure with the appropriate amount of rGO. The bare ZnS was prepared without the addition of rGO. The different wt% rGO-ZnS were utilized for NBB degradation under direct solar light and the results (Table S1, see Supplementary Materials) showed that 13.3 wt% rGO was found to be the optimum loading on ZnS for efficient removal of NBB. Hence, this catalyst was further characterized along with bare ZnS for comparison and utilized for further reactions.

## 3. Results

### 3.1. X-ray Diffraction Pattern of Reduced Graphene Oxide-ZnS

**Figure 1:**
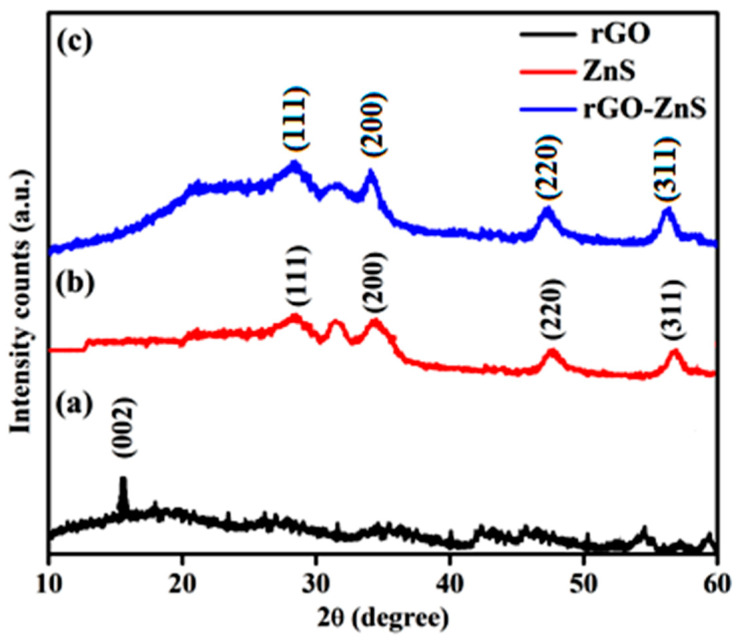
XRD patterns of (**a**) rGO, (**b**) ZnS, and (**c**) rGO-ZnS.

XRD was used to analyze the material’s structural nature. The rGO and rGO-ZnS XRD patterns are shown in Figure 1. The development of the small intensity diffraction peak 002 (plane) at 2*θ* = 15.06° demonstrates the presence of a low percentage of partly reduced graphene oxide (prGO) (Figure 1a) [46]. A broad peak in rGO that appears between 2*θ* = 20° and 30° is explained by the resuscitation of the van der Waals interaction following the reduction of graphene oxide [47]. As the graphene sheets are stacked in a limited number of layers, the broadness in the peak denotes the loss of long-range order [48]. Additionally, a reduction in the interlayer spacing reflects the loss of oxygen-containing groups and water molecules from the surface of graphene oxide. The diffraction peaks associated with 2*θ* = 28.56°, 33.5°, 47.2°, and 56.8° are seen in the ZnS (Figure 1b), which correspond to the cubic ZnS structure (JCPDS file no. 65-1691) planes (111), (200), (220) and (311) [49]. rGO-ZnS composites exhibit XRD patterns similar to bare ZnS, and no rGO diffraction peaks can be observed in the composites, which could be a result of the rGO’s low concentration and low diffraction intensity in the composite (Figure 1c).

### 3.2. Scanning Electron Microscope Analysis

**Figure 2:**
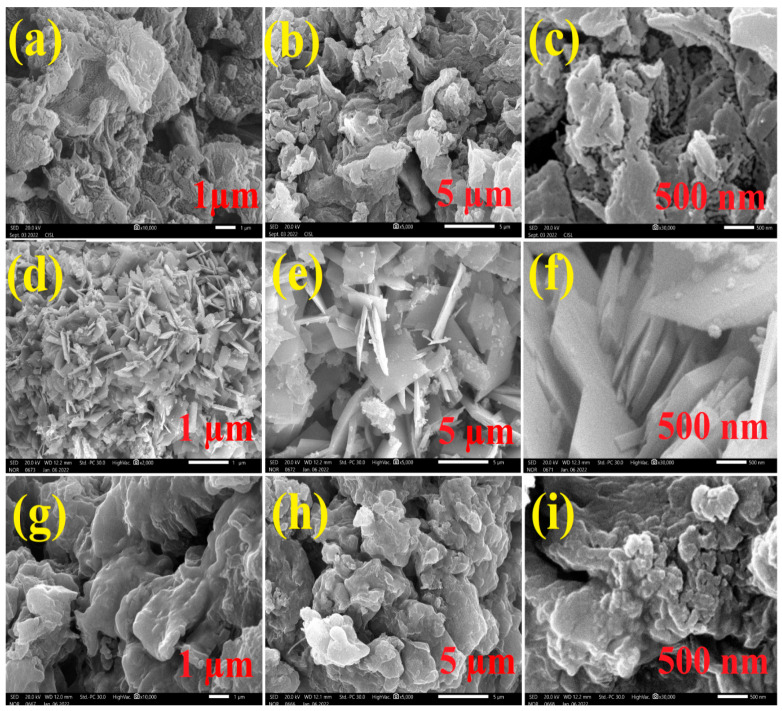
SEM images of (**a**–**c**) rGO, (**d**–**f**) ZnS and (**g**–**i**) rGO-ZnS.

SEM analysis was used to examine the precise morphology and microstructure of the catalysts. The SEM images of rGO, ZnS, and rGO-ZnS samples with different magnifications are shown in Figure 2a–i. The rGO depicts a few layered structures, some of which are nanosheets, as should be visible in SEM (Figure 2a–c) [50]. The SEM resemblance of ZnS is depicted as a layered sheet-like structure (Figure 2d–f) [51]. Figure 2g–i display the rGO-ZnS surface morphology of the particles, which represents a broccoli-like structure.

### 3.3. Transmission Electron Microscope Analysis

The resulting rGO-ZnS composites were further studied using a high-resolution transmission electron microscope (HR-TEM). The shape of the rGO sheet is shown in Figure S2a (See Supplementary Materials). ZnS nanoparticles have been built evenly and deposited on the surface of the rGO (Figure S2b). Furthermore, the homogeneous distribution of ZnS nanoparticles on rGO sheets can reduce the aggregation of ZnS nanoparticles and increase reactive sites, hence improving photocatalytic performance [52].

### 3.4. Energy Dispersive X-ray Analysis

As shown in Figure S3, the EDX spectra of the rGO, ZnS, and rGO-ZnS nanocomposite were collected concurrently with SEM. The characteristic peaks of carbon, oxygen, zinc, and sulfur can be detected without any other elements emerging, showing the elemental composition of the nanocomposite.

### 3.5. Fourier Transfer-Infra Red Spectra

**Figure 3:**
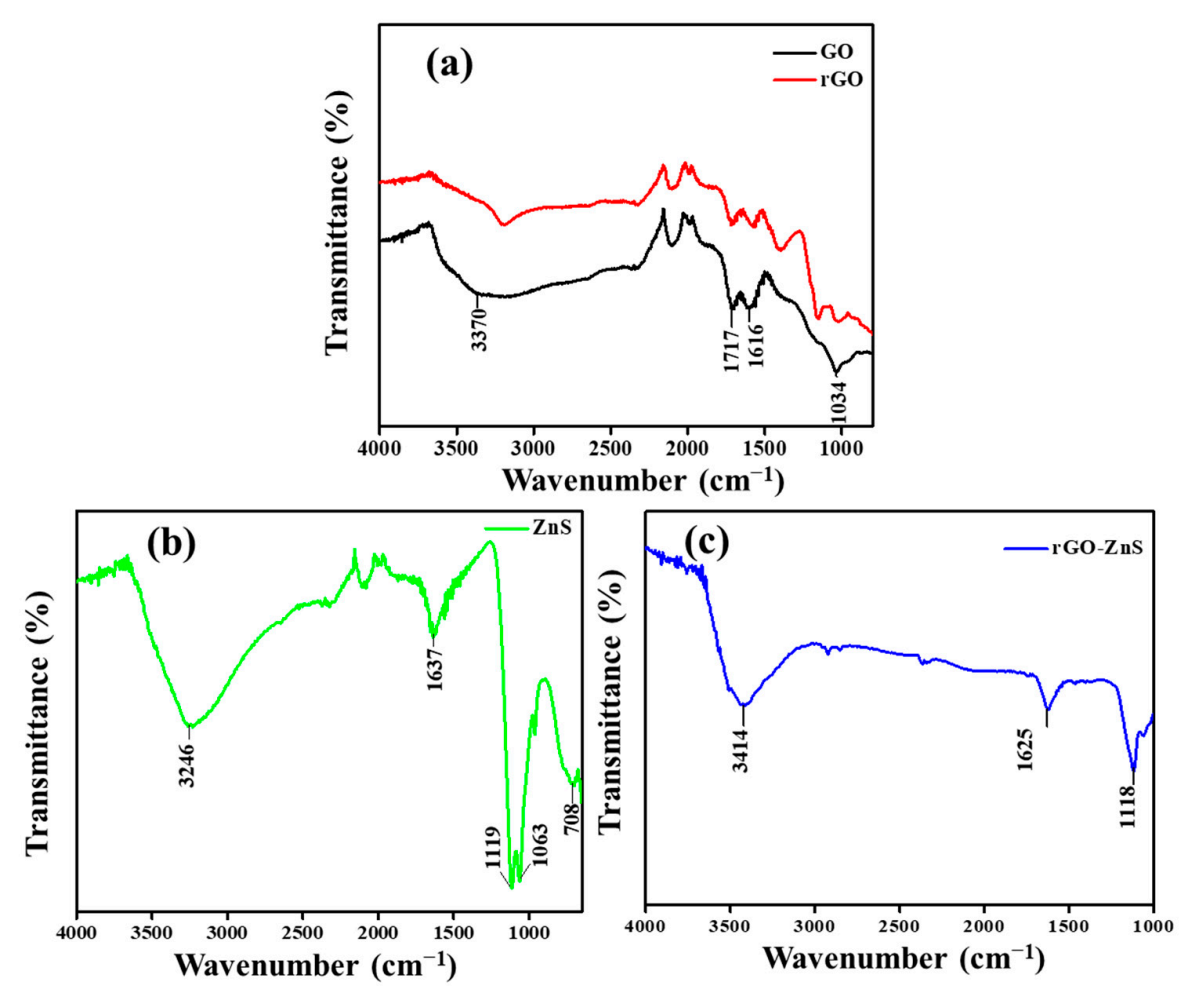
FT-IR spectra of (**a**) GO and rGO, (**b**) ZnS, and (**c**) rGO-ZnS.

Figure 3 displays the FT-IR spectra of GO, rGO, ZnS, and rGO-ZnS composite across the range of 500–4000 cm^−1^. In FT-IR spectra of GO, the absorption band at 1616 cm^−1^ is caused by skeletal vibrations of unoxidized graphitic domains (C=C stretching) [53]. The other peaks are due to stretching vibrations of –C=O (1717 cm^−1^) and C-O-C (1034 cm^−1^). The broad absorption band located at 3246 cm^−1^ can be attributed to the O-H stretching mode of H_2_O adsorbed on the surface of the particles in synthesized ZnS nanoparticles [54]. The stretching vibrations of the adsorbed H_2_O molecules found in GO are believed to be the cause of the intense and wide absorption at 3370 cm^−1^. The characteristic stretching -OH, C=O, and C-O are attributed to the absorption bands at 3414, 1625, and 1118 cm^−1^ for the rGO-ZnS composite, respectively. This indicates that there are still some oxygen-containing groups of GO sheets because the hydrothermal reduction of the absorption peaks in the rGO was significantly reduced, and some absorption peaks for C=O and C-O are absent [55].

### 3.6. X-ray Photoelectron Spectroscopy

**Figure 4:**
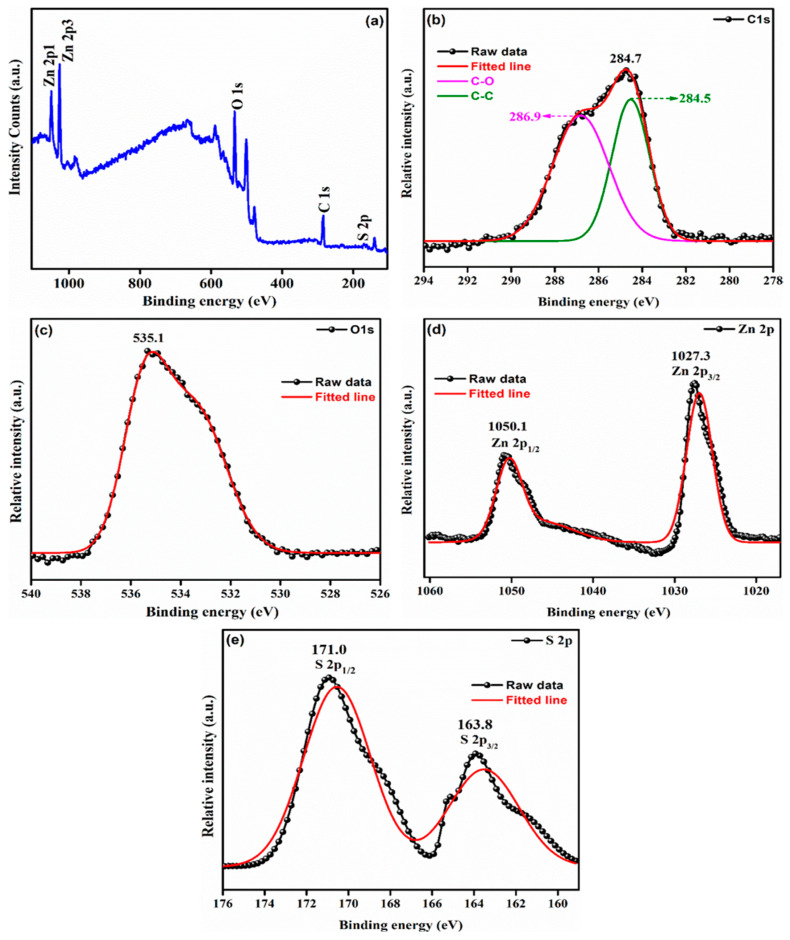
XPS spectra of the rGO-ZnS. (**a**) Survey spectrum of rGO-ZnS, (**b**) C1s, (**c**) O 1 s, (**d**) Zn 2p, and (**e**) S 2p.

The oxidation state of the elements present in rGO-ZnS is investigated using X-ray photoelectron spectroscopy (Figure 4). The presence of C, O, Zn, and S elements is confirmed via the XPS survey spectrum of the rGO-ZnS sample (Figure 4a). Figure 4b shows the C 1 s profile for rGO. Three strong peaks were observed at 284.5, 284.7, and 286.9 eV, which correlated to C-C, C-OH, and C-O, respectively [56]. The characteristic peaks of O 1 s were assigned a binding energy of 535.1 eV (Figure 4c). The typical peaks of Zn 2p_3/2_ and Zn 2p_1/2_ were assigned binding energies of 1027.3 eV and 1050.1 eV, revealing that the zinc ions were in the +2 oxidation state (Figure 4d). Binding energies of 163.8 eV and 171.0 eV were assigned to S 2p_3/2_ and 2p_1/2_, suggesting the presence of Zn-S bonds in the composite (Figure 4e) [57].

### 3.7. Photocatalytic Activity Studies

**Figure 5:**
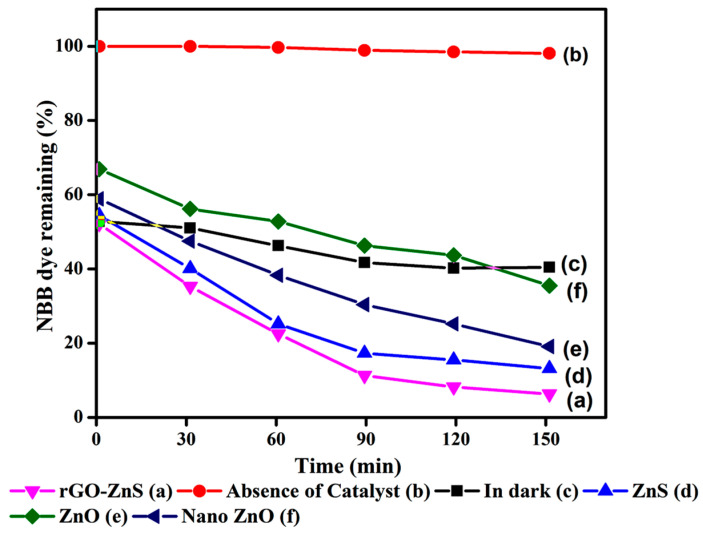
Photodegradability of NBB dye using solar/rGO-ZnS nanoparticles: [NBB] = 2 × 10^−4^ mol/L, pH = 7.0 ± 0.1, rGO-ZnS nanoparticles = 2 g/L, airflow rate = 8.1 mL s^−1^, *I*_solar_ = 1250 × 100 ± 100 lux.

**Figure 6:**
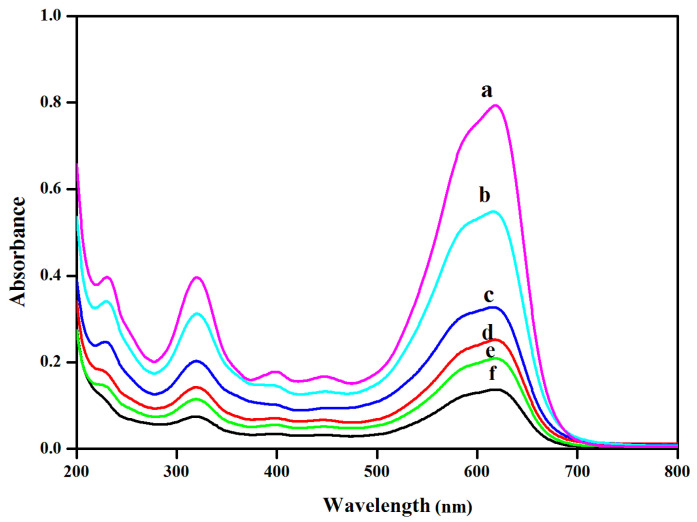
The changes in UV-visible spectra of NBB on irradiation with solar light in the presence of rGO-ZnS nanoparticles: [NBB] = 2 × 10^−4^ mol/L, pH = 7.0 ± 0.1, rGO-ZnS nanoparticles = 2 g/L, airflow rate = 8.1 mL s^−1^, *I*_solar_ = 1250 × 100 ± 100 lux. a = 0 min, b = 30 min, c = 60 min, d = 90 min, e = 120 min, and f = 150 min.

Figure 5 depicts the percentage of NBB dye that is still present after solar light is used to irradiate an aqueous solution of NBB (2 × 10^−4^ M). At 150 min under solar light, degradation of 93.7% of NBB occurs in the presence of rGO-ZnS (curve a). In the absence of a catalyst, negligible deterioration (0.2%) took place in the presence of solar irradiation (curve b). In contrast, the dye concentration decreased by 47.8% in an identical experiment using rGO-ZnS in the absence of solar radiation (curve c). This can be the result of the dye adhering to the catalyst’s surface. These observations reveal that sunlight and catalyst must be essential for the degradation of NBB dye. Under the same conditions, only 81.8 (curve d), 78.8 (curve e), and 61.1 (curve f) percent degradation occurred when other photocatalysts such as ZnS, ZnO, and Nano ZnO were utilized. This demonstrates that compared to other procedures, the rGO-ZnS approach is more effective at degrading NBB dye. The UV-vis spectra of NBB dye at different times of irradiation with rGO-ZnS is shown in Figure 6. There is no significant change in UV maxima during irradiation, but the intensities at 320 nm and 617 nm decrease gradually during degradation. This reveals that the intermediate does not absorb at the analytical wavelength of 320 nm and 617 nm.

#### 3.7.1. Effect of Catalyst Weight

Various studies have shown that catalyst dosage greatly influences reaction rate. We investigated how different photocatalyst dosages affected the photocatalytic degradation of naphthol blue black, and the results are presented in Figure S4. The dosage ranged between 1 and 3 g/L, and room temperature was used for all of the readings. It can be said that the degradation efficiencies were greatly increased at 90 min of irradiation with the increase in catalyst dosage from 1 to 2 g/L; after that, they decreased. This may be due to the presence of more active sites which enhances the rate of degradation (up to 2 g/L). The reason for the decrease in degradation at a particular level (beyond 2 g/L) may be due to the light scattering effect, which would render the penetration of light into the dye solution [58]. So, the experiment was carried out with a 2 g/L rGO-ZnS nanocomposite.

#### 3.7.2. Effect of pH

**Figure 7:**
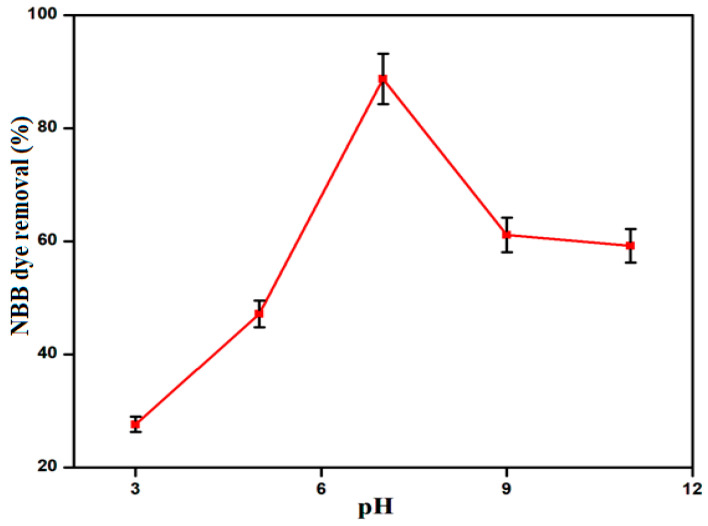
Effect of initial pH on the degradation of NBB using solar light/rGO-ZnS nanoparticles: [NBB] = 2 × 10^−4^ mol/L, rGO-ZnS nanoparticles = 2 g/L, airflow rate = 8.1 mL s^−1^, irradiation time = 90 min, *I*_solar_ = 1250 × 100 ± 100 lux.

The ionization state of the dye and the photocatalyst’s surface characteristics are both impacted by the pH, making it a crucial parameter in photocatalytic degradation. Changes in pH can also affect how pollutants adhere to the photocatalyst surface, which is necessary for photocatalytic oxidation to occur. Before radiation, the dye solution pH was adjusted, but it was not monitored throughout the reaction. Furthermore, the pH of the solution influences the surface charge characteristics of rGO-ZnS, the size of the aggregates formed, the surface charge of dye molecules, dye adsorption onto catalyst surfaces, and the level of hydroxyl radicals. The influence of pH on the photocatalytic degradation of NBB was investigated over a pH range of 3–11. The outcomes are displayed in Figure 7. According to the observations, neutral pH 7 exhibits the highest rGO-ZnS optimum efficiency. The NBB dye was dark-adsorption tested at various pH levels to ascertain the cause of this. Following the achievement of adsorption equilibrium, the initial dye adsorption rates observed at pH 3, 5, 7, 9, and 11 are 27.4, 38.1, 47.8, 35.3, and 24.6 percent, respectively. At pH 7, there was the greatest amount of adsorption. Since the adsorption is higher at pH 7, the breakdown is also effective at this pH.

#### 3.7.3. Effect of Initial Dye Concentration

Investigations have been conducted into the impact of different initial concentrations of the NBB dye on its degradation by the rGO-ZnS catalyst (Figure S5). Degradation is reduced from 95.1% to 42.8% as dye concentration rises from 1 to 4 × 10^−4^ mol/L. The rate of deterioration is dependent on the generation of ^•^OH on the catalyst surface and the likelihood that these radicals will interact with dye molecules. The catalyst dosage and light output are the same for all initial dye concentrations. So, the limited number of hydroxyl radicals (^•^OH) available for all the dye concentrations thus decreases the degradation efficiency. Moreover, at higher concentrations, the path length of entering photons is significantly reduced.

#### 3.7.4. Radical Scavengers Test

The influence of different radical scavengers such as ethanol, isopropyl alcohol (IPA), and benzoquinone (BQ) on photocatalytic degradation is investigated and shown in Table 1. Without the addition of scavengers, photocatalytic degradation is significantly accelerated (88.7%), as demonstrated in Table 1. When isopropyl alcohol is added, degradation is reduced (63.5%) because the scavenger (isopropyl alcohol) removes the hydroxyl radicals (^•^OH), and the degradation rate is also reduced. Ethanol and benzoquinone were employed for removing h^+^ (holes) and superoxide (O_2_^•−^) radical anions, respectively. The addition of ethanol and benzoquinone decreases degradation, which was found to be 52.1 and 63.5%, respectively. All the scavengers decrease the degradation efficiency of the catalyst, and the addition of benzoquinone significantly reduces the degradation efficiency, revealing that superoxide (O_2_^•−^) radical anions are active main species in the degradation process followed by h^+^ (holes) and hydroxyl radicals (^•^OH) [59].

#### 3.7.5. Stability of the Catalyst

**Figure 8:**
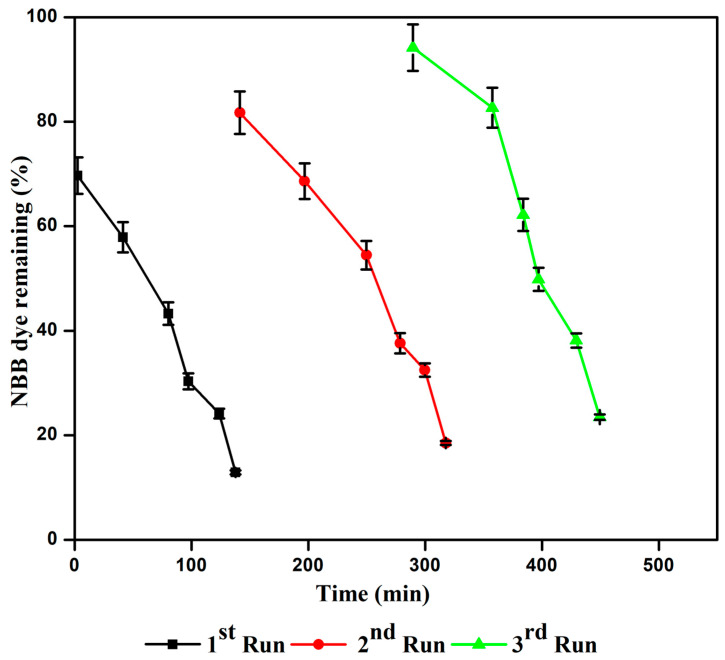
Catalyst reusability. [NBB] = 2 × 10^−4^ mol/L, pH = 7.0 ± 0.1, rGO-ZnS nanoparticles = 2 g/L, airflow rate = 8.1 mL s^−1^, *I*_solar_ = 1250 × 100 ± 100 lux.

In the same conditions, the NBB dye was used to test the recyclability of rGO-ZnS. The effects are shown in Figure 8. The first, second, and third runs’ degradation efficiencies are 93.7, 68.4, and 63.9%, respectively. It demonstrates that throughout three runs, rGO-ZnS displayed outstanding photostability without suffering a sizable decrease in photocatalytic activity. These results indicate that under solar light, the rGO-ZnS catalyst is still effective and recyclable.

#### 3.7.6. Kinetic Analysis and Literature Comparison

rGO-ZnS containing naphthol blue black undergoes heterogeneous photocatalytic degradation that reportedly follows pseudo-first-order kinetics. The rate determination is given via Equation (1) at low beginning substrate (NBB) concentrations.

(1) $$\frac{-d\left[C\right]}{dt}=k^{\prime }\left[C\right]$$

where the pseudo-first-order rate constant is denoted by *k′*. Table S2 provides the deterioration rate constant $\left(k^{\prime }\right)$. The equilibrium between adsorption and desorption is achieved once naphthol blue black is adsorbed onto the rGO-ZnS surface. The equilibrium concentration of the naphthol blue black solution is discovered after adsorption, and it is used as the starting concentration of naphthol blue black for kinetic analysis. The following equation is obtained through integrating the previously mentioned equation with the limit of *C* = *C*_0_ at time *t* = 0, where *C*_0_ is the equilibrium concentration of the bulk solution.

(2) $$ln\left[\;C_{0}/C\right]=k^{\prime }t$$

where *C*_0_ is the naphthol blue black equilibrium concentration and *C* is the concentration at time *t*.

**Figure 9:**
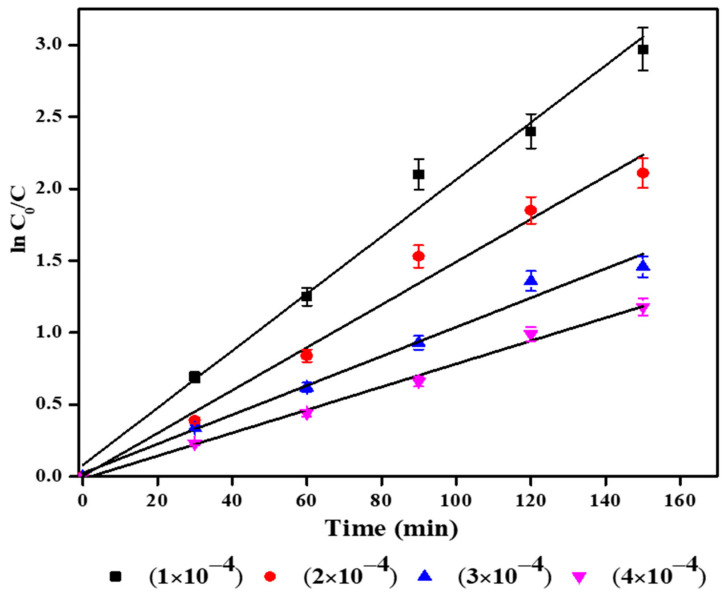
Kinetics of NBB dye degradation for different initial concentrations via solar light/rGO-ZnS nanoparticles.

**Figure 10:**
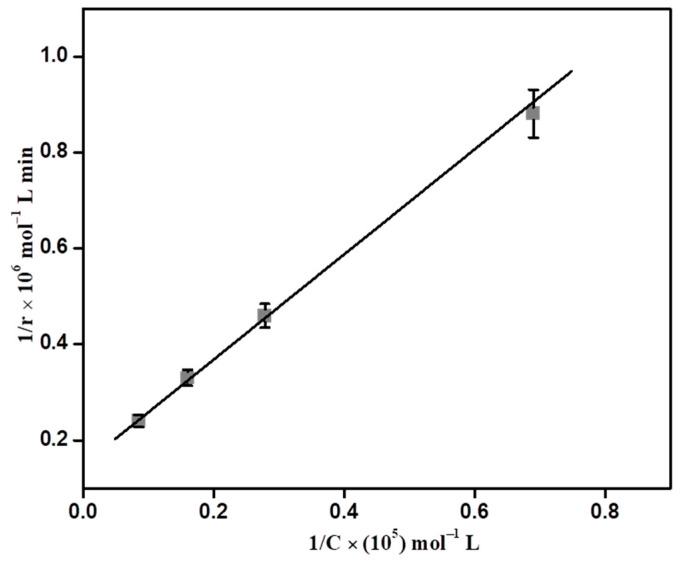
A linearized reciprocal kinetic plot of the degradation of NBB via solar light/rGO-ZnS nanoparticles.

Figure 9 displays a plot of ln *C*_0_/*C* vs. time for deterioration. It has been discovered that the relationship between naphthol blue black concentration and exposure time is linear. The Langmuir–Hinshelwood (L-H) kinetic expression has been effectively employed by several authors to investigate the heterogeneous photocatalytic reaction. To explain the solid–liquid reaction, the experimental data have been justified using a modified version of the L-H kinetic model. Assuming that naphthol blue black is more strongly adsorbed on the catalytic surface than the intermediate products, the rate of oxidation of naphthol blue black at the surface reaction is proportional to the surface coverage of naphthol blue black on rGO-ZnS. Equations (3) and (4) are used to show how the concentration of naphthol blue black influences the rate of deterioration [60].

(3) $$r={\;K}_{1}K_{2}C\;/\;1+K_{1}C\;$$

(4) $$\frac{1}{r}=\frac{1}{K_{2}K_{1}C}+\frac{1}{K_{2}}\;$$

where K_1_ is the constant linked to adsorption, K_2_ is related to the reaction characteristics of the substrate NBB, and “*C*” denotes the concentration of NBB at the time “*t*”. The linear plot was constructed through graphing the reciprocal of the initial rate (1/*r*) against the reciprocal of the initial concentration NBB (1/C), as shown in Figure 10. It has been used to confirm the L-H equation’s applicability for degradation. The slope and intercept of these plots have been used to get the values of K_1_ and K_2_. For deterioration, K_1_ and K_2_ are determined to have values of 1.318 × 10^3^ M^−1^ and 2.120 × 10^−5^ Mm^−1^, respectively. The photodegradation efficiency of rGO-ZnS was compared with different modified ZnS materials, and the results (Table S3) showed that rGO-ZnS efficiently degrades NBB dye under solar light.

#### 3.7.7. COD Measurements

COD values were also used to confirm the mineralization (degradation) of NBB dye. Table 2 provides the percentage of COD decreases. After 150 min of rGO-ZnS irradiation, 91.1% COD removal is attained. This shows that the mineralization of the dye is almost completed [61].

#### 3.7.8. Mechanism of Degradation

**Scheme 1:**
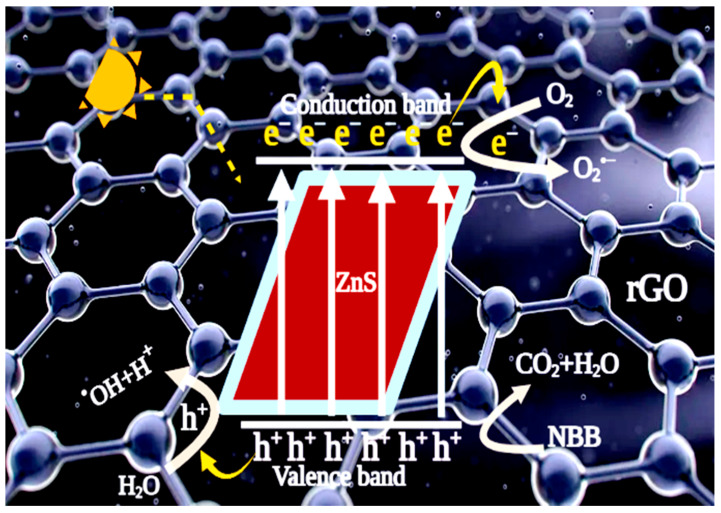
Mechanism of NBB dye degradation using rGO-ZnS.

Solar light is used to illuminate the modified semiconductor (rGO-ZnS), resulting in the generation of electron–hole pairs, holes from the valence band, and electrons from the conduction band (Scheme 1). Conduction band electrons are more likely to move to rGO in the modified ZnS nanocomposite, which is widely recognized as an efficient electron acceptor and transporter in nanocomposites during the photocatalytic process (rGO-ZnS). The photogenerated holes can be captured by hydroxyl or water molecules to produce highly active ^•^OH species, which deteriorate the pollutant (dye) [62]. The dissolved oxygen molecule absorbs the conductance band electron, producing an active superoxide radical anion (O_2_^•−^). In an aqueous environment, O_2_^•−^ produces other highly reactive species such as HO_2_^•^ (hydroperoxide radical), HO^•^ (hydroxyl radical), and H_2_O_2,_ which also oxidizes organic compounds (Equations (5)–(10)) [63].

rGO-ZnS + hν → h^+^ + e^−^ (5)

h^+^ + H_2_O →^•^OH + H^+^ (6)

O_2_ + e^−^ → O_2_^•−^ (7)

O_2_^•−^ + H^+^ → HO_2_^•^ (8)

O_2_^•−^ + NBB dye → Degraded products (Mineral acids + CO_2_ + H_2_O) (9)

^•^OH + NBB dye → Degraded products (Mineral acids + CO_2_ + H_2_O) (10)

## 4. Antibacterial Studies

**Figure 11:**
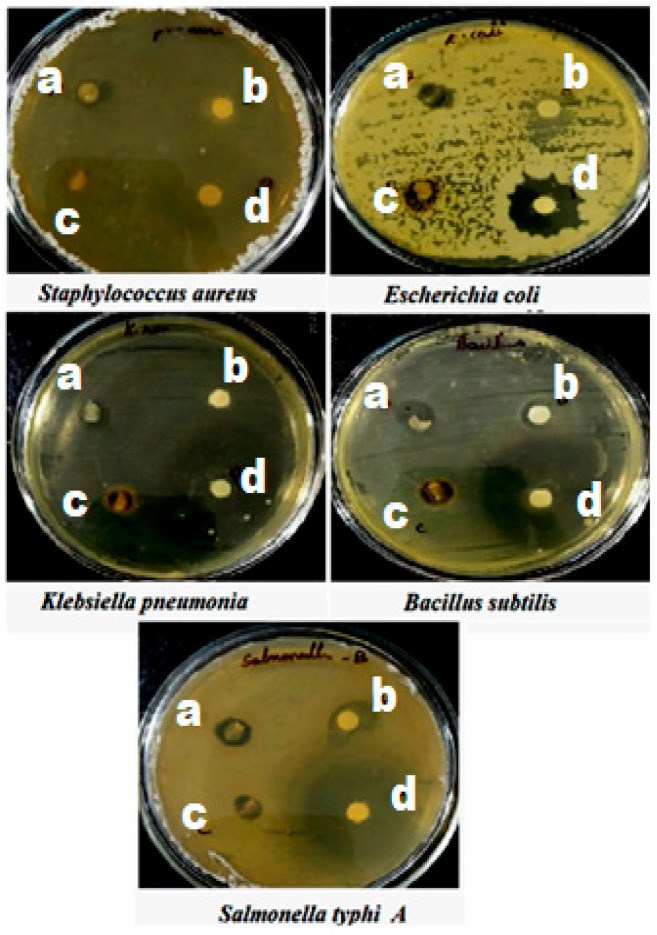
Antibacterial activities of (a) rGO (b) ZnS (c) standard pharmaceutical (Amoxillin) and (d) rGO-ZnS.

The disc diffusion method was adopted for the elevation of antimicrobial activity. This test employs Gram-positive bacteria such as Bacillus subtilis and Staphylococcus aureus, as well as Gram-negative bacteria such as Escherichia coli, Salmonella typhi A, and Klebsiella pneumonia. The control is a filter paper that does not contain any nanocomposite. Standard treatment involves the administration of amoxicillin. Test pathogens were propagated on agar plates during the antibacterial measurement. All of the wells were made using a sterile cork borer, loaded with the necessary amount of nanocatalyst (rGO, ZnS, and rGO-ZnS), and then put over the agar. The test plates were incubated for 24 h at 37 °C. Activity against the test pathogens was determined through measuring the zone of inhibition (mm in diameter). One well was for the standard (amoxicillin) and three other wells were for other nanomaterials (rGO, ZnS, and rGO-ZnS) (Figure 11), and the values are given in Table 3. rGO and ZnS alone have insufficient activity against all of the investigated bacterial strains, as demonstrated in Figure 11. The rGO-ZnS nanocomposite was found to have superior antibacterial activity compared to the antibacterial activities of rGO and ZnS (Table 3). rGO-ZnS has better activity against Bacillus subtilis, Salmonella typhi, and Klebsiella pneumonia than other micro-organisms.

## 5. Conclusions

A novel rGO containing ZnS was created using a solvothermal technique. Under solar light, it was discovered that rGO-ZnS is more effective than ZnS at deteriorating NBB azo dye, and rGO-ZnS has better activity against Bacillus subtilis, Salmonella typhi, and Klebsiella pneumonia than other micro-organisms. A pH of 7 and a catalyst concentration of 2 g/L were found to be optimal for effective dye removal. The degradation rate was reduced as the initial dye concentration was increased. The stability of the catalyst was revealed. This approach is more cost-effective and environmentally beneficial owing to the wastewater treatment technique. COD (91.1% removal) observations indicated that the NBB molecule had been mineralized. A mechanism explains that rGO-ZnS has superior photocatalytic activity for NBB degradation under solar light. Since solar operations only require a small amount of catalyst (2 g/L), this catalyst will be extremely effective for the solar treatment of dye effluent.

## Figures and Tables

**Table 1:** Effect of different radical scavengers on photodegradation of NBB dye with rGO-ZnS under solar light.

Different Radical Scavengers	NBB Dye Degradation Percentage (%)
No Scavengers	88.7
Isopropyl alcohol	63.5
Ethanol	52.1
Benzoquinone	38.2

[NBB] = 2 × 10^−4^ mol/L, Catalyst suspended = 2 g/L, pH = 9, irradiation time = 90 min, [scavengers] = 5 mmol.

**Table 2:** COD values and removal percentage on the photocatalytic degradation of NBB dye using solar light with rGO-ZnS.

Time (min)	COD Values (mg/L)	COD Removal (%)
0	9049.6	0
90	5376.0	59.4
150	806.4	91.1

COD measurements: [NBB] = 2 × 10^−4^ mol/L, rGO-ZnS nanoparticles suspended 2 g/L, pH = 7, airflow rate = 8.1 mL s^−1^.

**Table 3:** Antibacterial zone of inhibition capacities owing the prepared samples of rGO, ZnS, and rGO-ZnS with different bacterial strains.

Serial No.	Microbial Strains	Zone of Inhibition (mm)			
		Standard (30 µL)	rGO (30 µL)	ZnS (30 µL)	rGO-ZnS (30 µL)
	**Gram Positive (+)**				
1	*Staphylococcus aureus*	30	9	9	15
2	*Bacillus subtilis*	30	11	12	21
	**Gram negative (−)**				
3	*Salmonella paratyphi A*	22	10	10	23
4	*Escherichia coli*	20	11	9	17
5	*Klebsiella pneumonia*	32	10	9	19

## Data Availability

The datasets used or analyzed during the current study are available from the corresponding author upon reasonable request.

## Supplementary Materials

The following supporting information can be downloaded at: https://www.mdpi.com/article/10.3390/mi14071324/s1, Figure S1: Chemical structure of NBB dye; Figure S2: HR-TEM images of (a) rGO (b) rGO-ZnS; Figure S3: EDX spectra of (a) rGO (b) ZnS (c) rGO-ZnS; Figure S4: Effect of catalyst weight on the photocatalytic degradation of NBB using solar light; Figure S5: Effect of various initial dye concentrations on the degradation of NBB using solar light/rGO-ZnS nanoparticles; Table S1: Effect of different wt% of rGO on ZnS for the NBB dye degradation under solar light; Table S2: Rate constants of photocatalytic degradation of NBB dye using solar light/rGO-ZnS nanoparticles; Table S3: Comparison of different modified ZnS towards pollutant degradation [64,65,66,67,68].

## References

[1] Shen Y., Li L., Xiao K., Xi J. (2016). Constructing Three-Dimensional Hierarchical Architectures by Integrating Carbon Nanofibers into Graphite Felts for Water Purification. ACS Sustain. Chem. Eng..

[2] Zhang P., Li J., Lv L., Zhao Y., Qu L. (2017). Vertically Aligned Graphene Sheets Membrane for Highly Efficient Solar Thermal Generation of Clean Water. ACS Nano..

[3] Guo K., Gao B., Tian B.X., Yue Q., Zhang P., Shen X., Xu X. (2019). Synthesis of polyaluminium chloride/papermaking sludge-based organic polymer composites for removal of disperse yellow and reactive blue by flocculation. Chemosphere.

[4] Amanulla B., Sannasi S., Abubakker A.K.M., Ramaraj S.K. (2018). A magnetically recoverable bimetallic Au-FeNPs decorated on g-C_3_N_4_ for efficient photocatalytic degradation of organic contaminants. J. Mol. Liq..

[5] Colmenares J.C., Luque R. (2014). Heterogeneous photocatalytic nanomaterials: Prospects and challenges in selective transformations of biomass-derived compounds. Chem. Soc. Rev..

[6] Bhatkhande D.S., Pangarkar V.G., Beenackers A.A.C.M. (2002). Photocatalytic degradation for environmental applications—A review. J. Chem. Technol. Biotechnol..

[7] Jing L., Zhou W., Tian G., Fu H. (2013). Surface tuning for oxide-based nanomaterials as efficient photocatalysts. Chem. Soc. Rev..

[8] Kuzhalosai V., Subash B., Senthilraja A., Dhatshanamurthi P., Shanthi M. (2013). Synthesis, characterization and photocatalytic properties of SnO_2_–ZnO composite under UV-A light. Spectrochim. Acta A Mol. Biomol. Spectrosc..

[9] Mohapatra L., Parida K. (2016). A review on the recent progress, challenges and perspective of layered double hydroxides as promising photocatalysts. J. Mater. Chem. A.

[10] Liu B., Zhao X., Terashima C. (2014). Thermodynamic and kinetic analysis of heterogeneous photocatalysis for semiconductor systems. Phys. Chem. Chem. Phys..

[11] Guo Q., Zhou C., Ma Z., Ren Z., Fan H., Yang X. (2016). Elementary photocatalytic chemistry on TiO_2_ surfaces. Chem. Soc. Rev..

[12] Liu L., Liu J., Sun D.D. (2012). Graphene oxide enwrapped Ag_3_PO_4_ composite: Towards a highly efficient and stable visible-light-induced photocatalyst for water purification. Catal. Sci. Technol..

[13] Xiang X., Xie L., Li Z., Li F. (2013). Ternary MgO/ZnO/In_2_O_3_ heterostructured photocatalysts derived from a layered precursor and visible-light-induced photocatalytic activity. Chem. Eng. J..

[14] Dhatshanamurthi P., Subash B., Senthilraja A., Kuzhalosai V., Krishnakumar B., Shanthi M. (2014). Synthesis and Characterization of ZnS–TiO_2_ Photocatalyst and Its Excellent Sun Light Driven Catalytic Activity. J. Nanosci. Nanotechnol..

[15] Yan Z., Sun Z., Liu X., Jia H., Du P. (2016). Cadmium sulfide/graphitic carbon nitride heterostructure nanowire loading with a nickel hydroxide cocatalyst for highly efficient photocatalytic hydrogen production in water under visible light. Nanoscale.

[16] Jiang W., Liu Y., Zong R., Li Z., Yao W., Zhu Y. (2015). Photocatalytic hydrogen generation on bifunctional ternary heterostructured In_2_S_3_/MoS_2_/CdS composites with high activity and stability under visible light irradiation. J. Mater. Chem. A.

[17] Khanchandani S., Kundu S., Patra A., Ganguli A.K. (2012). Shell Thickness Dependent Photocatalytic Properties of ZnO/CdS. J. Phys. Chem. C.

[18] Li N., Zhang L., Zhou J., Jing D., Sun Y. (2014). Localized nano-solid-solution induced by Cu doping in ZnS for efficient solar hydrogen generation. Dalt. Trans..

[19] Zhu T., Zhang C., Ho G.W. (2015). In situ dissolution-diffusion toward homogeneous multiphase Ag/Ag_2_S@ZnS core-shell heterostructures for enhanced photocatalytic performance. J. Phys. Chem. C.

[20] Han L., Wang P., Dong S. (2012). Progress in graphene-based photoactive nanocomposites as a promising class of photocatalyst. Nanoscale.

[21] Tang Y., Wang R., Yang Y., Yan D., Xiang X. (2016). Highly Enhanced Photoelectrochemical Water Oxidation Efficiency Based on Triadic Quantum Dot/Layered Double Hydroxide/BiVO_4_ Photoanodes. ACS Appl. Mater. Interfaces.

[22] Li Y., Zhang L., Xiang X., Yan D., Li F. (2014). Engineering of ZnCo-layered double hydroxide nanowalls toward high-efficiency electrochemical water oxidation. J. Mater. Chem. A.

[23] Lakshmipathy R., Kesarla M.K., Nimmala A.R., Godavarthi S., Kukkambakam C.M., Gomez L.M., Sarada N.C. (2017). ZnS nanoparticles capped with watermelon rind extract and their potential application in dye degradation. Res. Chem. Intermed..

[24] Wang R., Pan K., Han D., Jiang J., Xiang C., Huang Z., Zhang L., Xiang X. (2016). Solar-Driven H_2_O_2_ Generation From H_2_O and O_2_ Using Earth-Abundant Mixed-Metal Oxide@Carbon Nitride Photocatalysts. ChemSusChem.

[25] He W., Wang R., Zhang L., Zhu J., Xiang X., Li F. (2015). Enhanced photoelectrochemical water oxidation on a BiVO_4_ photoanode modified with multi-functional layered double hydroxide nanowalls. J. Mater. Chem. A.

[26] Wei X.X., Chen C.M., Guo S.Q., Guo F., Li X.M., Wang X.X., Cui H.T., Zhao L.F., Li W. (2014). Advanced visible-light-driven photocatalyst BiOBr-TiO_2_-graphene composite with graphene as a nano-filler. J. Mater. Chem. A.

[27] Wu H., Wang Q., Yao Y., Qian C., Zhang X., Wei X. (2008). Microwave-assisted synthesis and photocatalytic properties of carbon nanotube/zinc sulfide heterostructures. J. Phys. Chem. C.

[28] Ye A., Fan W., Zhang Q., Deng W., Wang Y. (2012). CdS-graphene and CdS-CNT nanocomposites as visible-light photocatalysts for hydrogen evolution and organic dye degradation. Catal. Sci. Technol..

[29] Akcöltekin S., El Kharrazi M., Köhler B., Lorke A., Schleberger M. (2009). Graphene on insulating crystalline substrates. Nanotechnology.

[30] Reina A., Jia X., Ho J., Nezich D., Son H., Bulovic V., Dresselhaus M.S., Kong J. (2009). Large Area, Few-Layer Graphene Films on Arbitrary Substrates by Chemical Vapor Deposition. Nano Lett..

[31] Borovikov V., Zangwill A. (2009). Step-edge instability during epitaxial growth of graphene from SiC(0001). Phys. Rev. B-Condens. Matter Mater. Phys..

[32] Shin B.H., Kim K.K., Benayad A., Yoon S., Park K., Jung I., Jin M.H., Jeong H., Kim J.M., Choi J. (2009). Efficient Reduction of Graphite Oxide by Sodium Borohydride and Its Effect on Electrical Conductance. Adv. Funct. Mater..

[33] Khamboonrueang D., Srirattanapibula S., Tang I.-M., Thongmeea S. (2018). TiO_2_∙rGO nanocomposite as a photo catalyst for the reduction of Cr^6+^. Mater. Res. Bull..

[34] Yuan J., Lu Y., Huang B., Xu H., Tao Y., Xu H., Zhang W., He G., Chen H. (2022). Al-doping driven electronic structure of α-NiS hollow spheres modified by rGO as high-rate electrode for quasi-solid-state capacitor. Ceram. Int..

[35] Srirattanapibul S., Tang I.-M., Thongmee S. (2020). Photo catalytic reduction of Cr^6+^ by ZnO decorated on reduced graphene oxide (rGO) Nanocomposites. Mater. Res. Bull..

[36] Imboon T., Khumphon J., Yotkuna K., Tang I.-M., Thongmee S. (2021). Enhancement of photocatalytic by Mn_3_O_4_ spinel ferrite decorated graphene oxide nanocomposites. SN Appl. Sci..

[37] Liu W., Cai J., Li Z. (2015). Self-Assembly of Semiconductor Nanoparticles/Reduced Graphene Oxide (RGO) Composite Aerogels for Enhanced Photocatalytic Performance and Facile Recycling in Aqueous Photocatalysis. ACS Sustain. Chem. Eng..

[38] Nawaz M., Islam M.U., Nazir M.A., Bano I., Gul I.H., Ajmal M. (2021). Transport properties in spinel ferrite/graphene oxide nanocomposites for electromagnetic shielding. Ceram. Int..

[39] Lee J.S., You K.H., Park C.B. (2012). Highly Photoactive, Low Bandgap TiO_2_ Nanoparticles Wrapped by Graphene. Adv Mater..

[40] An X., Yu J.C. (2011). Graphene-based photocatalytic composites. RSC Adv..

[41] Wang C., Wang Y., Yang Z., Hu N. (2021). Review of recent progress on graphene-based composite gas sensors. Ceram. Int..

[42] Das T.K., Ghosh S.K., Das N.C. (2023). Green synthesis of a reduced graphene oxide/silver nanoparticles-based catalyst for degradation of a wide range of organic pollutants. Nano-Struct. Nano-Objects.

[43] Krishnakumar B., Subash B., Swaminathan M. (2012). AgBr–ZnO—An efficient nano-photocatalyst for the mineralization of Acid Black 1 with UV light. Sep. Purif. Technol..

[44] Chen J., Yao B., Li C., Shi G. (2013). An improved Hummers method for eco-friendly synthesis of graphene oxide. Carbon.

[45] Hou C., Zhang Q., Zhu M., Li Y., Wang H. (2011). One-step synthesis of magnetically-functionalized reduced graphite sheets and their use in hydrogels. Carbon.

[46] Lee M., Balasingam S.K., Jeong H.Y., Hong W.G., Lee H., Kim B.H., Jun Y. (2015). One-step hydrothermal synthesis of graphene decorated V_2_O_5_ nanobelts for enhanced electrochemical energy storage. Sci. Rep..

[47] Muthoosamy K., Geetha Bai R., Abubakar I.B., Sudheer S.M., Lim H.N., Loh H.S., Huang N.M., Chia C.H., Manickam S. (2015). Exceedingly biocompatible and thin-layered reduced graphene oxide nanosheets using an eco-friendly mushroom extract strategy. Int. J. Nanomed..

[48] De Silva K.S.B., Gambhir S., Wang X.L., Xu X., Li W.X., Officer D.L., Wexler D., Wallace G.G., Dou S.X. (2012). The effect of reduced graphene oxide addition on the superconductivity of MgB_2_. J. Mater. Chem..

[49] Phuruangrat A., Karthik K., Kuntalue K., Dumrongrojthanath P., Thongtem S., Thongtem T. (2019). Refuxing synthesis and characterization of ZnS nanoparticles and their photocatalytic properties. Chalcogenide Lett..

[50] Ramkumar R., Minakshi Sundaram M. (2016). A biopolymer gel-decorated cobalt molybdate nanowafer: Effective graft polymer cross-linked with an organic acid for better energy storage. R. Soc. Chem..

[51] Yu D., Fang H., Qiu P., Meng F., Liu H., Wang S., Lv P., Cong X., Niu Q., Li T. (2021). Improving the Performance of ZnS Photocatalyst in Degrading Organic Pollutants by Constructing Composites with Ag_2_O. Nanomater.

[52] Bai Y., Li J.P., Wang J., Liu L. (2017). Facile preparation 3D ZnS nanospheresreduced graphene oxide composites for enhanced photodegradation of norfloxacin. J. Alloys Compd..

[53] Nethravathi C., Rajamathi M. (2008). Chemically modified graphene sheets produced by the solvothermal reduction of colloidal dispersions of graphite oxide. Carbon.

[54] Labiah H., Lahbib K., Hindouri S., Touil S., Chaabane T.B. (2016). Insight of ZnS nanoparticles contribution in different biological uses. Asian Pac. J. Trop. Med..

[55] Feng Y., Feng N., Zhang G., Du G. (2014). One-pot hydrothermal synthesis of ZnS–reduced graphene oxide composites with enhanced photocatalytic properties. Cryst. Eng. Comm..

[56] Zhang S., Yang J., Chen B., Guo S., Li J., Li C. (2017). One-step hydrothermal synthesis of reduced graphene oxide/zinc sulfide hybrids for enhanced tribological properties of epoxy coatings. Surf. Coat. Technol..

[57] Pan S., Liu X. (2012). ZnS-Graphene nanocomposite: Synthesis, characterization and optical properties. J. Solid State Chem..

[58] Medidi S., Markapurapu S., Kotupalli M.R., Chinnam R.K.R., Susarla V.M., Gandham H.B., Sanasi P.D. (2018). Visible Light Photocatalytic Degradation of Methylene Blue and Malachite Green Dyes with CuWO_4_-GO Nano Composite. Mod. Res. Catal..

[59] Jayamani G., Shanthi M. (2020). An efficient nanocomposite CdS-ZnWO_4_ for the degradation of Naphthol Green B dye under UV-A light illumination. Nano-Struct. Nano-Objects.

[60] Rajamanickam D., Shanthi M. (2016). Photocatalytic degradation of an organic pollutant by zinc oxide–solar process. Arab. J. Chem..

[61] Baral A., Das D.P., Minakshi M.G., Ghosh M.K., Padhi D.K. (2016). Probing Environmental Remediation of RhB Organic Dye Using a-MnO_2_ under Visible-Light Irradiation: Structural, Photocatalytic and Mineralization Studies. Chem. Sel..

[62] Karunakaran C., Dhanalakshmi R. (2008). Photocatalytic performance of particulate semiconductors under natural sunshine—Oxidation of carboxylic acids. Sol. Energy Mater. Sol. Cells.

[63] Senthilraja A., Subash B., Krishnakumar B., Swaminathan M., Shanthi M. (2014). Novel Sr–Au–ZnO: Synthesis, characterization and photocatalytic activity. Superlattices Microstruct..

[64] Sadollahkhani A., Nur O., Willander M., Kazeminezhad I., Khranovskyy V., Eriksson M.O., Yakimova R., Holtz P.O. (2015). A detailed optical investigation of ZnO@ZnS core–shell nanoparticles and their photocatalytic activityat different pH values. Ceram. Int..

[65] Palanisamy G., Bhuvaneswari K., Bharathi G., Pazhanivel T., Grace A.N., Pasha S.K.K. (2021). Construction of magnetically recoverable ZnS@WO_3_@CoFe_2_O_4_ nanohybrid enriched photocatalyst for the degradation of MB dye under visible light irradiation. Chemosphere.

[66] Janbandhu S.Y., Suhaila C.T., Munishwar S.R., Jayaramaiah J.R., Gedam R.S. (2022). Borosilicate glasses containing CdS/ZnS QDs: A heterostructured composite with enhanced degradation of IC dye under visible-light. Chemosphere.

[67] Kokilavani S., Al-Farraj S.A., Thomas A.M., El-Serehy H.A., Raju L.L., Sudheer Khan S. (2021). Enhanced visible light driven photocatalytic and antibacterial activities of Ag_2_WO_4_ decorated ZnS nanocomposite. Ceram. Int..

[68] Liu D., Gong J., Sun C., Xu L., Song Y. (2022). Enhanced photocatalytic activity for degradation of ofloxacin and dye by hierarchical flower-like ZnS/MoS_2_/Bi_2_WO_6_ heterojunction: Synergetic effect of 2D/2D coupling interface and solid sulfide solutions. Catal. Commun..

