# Supplementary material for: Synthesis of Solar Light Active Reduced Graphene Oxide-ZnS Nanomaterial for Photocatalytic Degradation and Antibacterial Applications
Source: Micromachines (Basel). 2023 Jun 28;14(7):1324. doi: 10.3390/mi14071324 (PMC10386591; doi:10.3390/mi14071324)
Supplement: Supplementary file 1 [file micromachines-14-01324-s001.zip › micromachines-2447908-supplementary.pdf]

Supporting information's on

Synthesis of solar light active reduced graphene oxide-ZnS nanomaterial for photocatalytic degradation and antibacterial applications

B. Sathya priya[1], Kanakaraj Aruchamy[2,*], Tae Hwan Oh[2], Balakrishna Avula[3], Imran Hasan[4], and M. Shanthi[1,*]

[1]Department of chemistry, Annamalai University, Annamalainagar 608 002, Tamil Nadu, India.

[2]School of Chemical Engineering, Yeungnam University, Gyeongsan 38436, Republic of Korea.

[3]Department of Chemistry, Rajeev Gandhi Memorial College of Engineering and Technology (Autonomous), Nandyal-518501, India.

[4]Department of Chemistry, College of Science, King Saud University, Riyadh-11451, Saudi Arabia.

*Corresponding authors:

Prof. Dr. M. Shanthi,

Professor,

Department of chemistry, Annamalai University,

Annamalainagar 608 002,

Tamil Nadu, India

E-mail: shanthimsm@gmail.com (Prof. Dr. M. Shanthi)

E-mail: a.kanakaraj@yu.ac.kr (Dr. Kanakaraj Aruchamy)

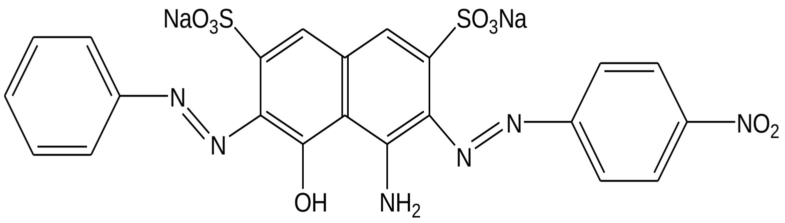

Figure S1. Chemical Structure of Naphthol Blue Black dye (NBB)

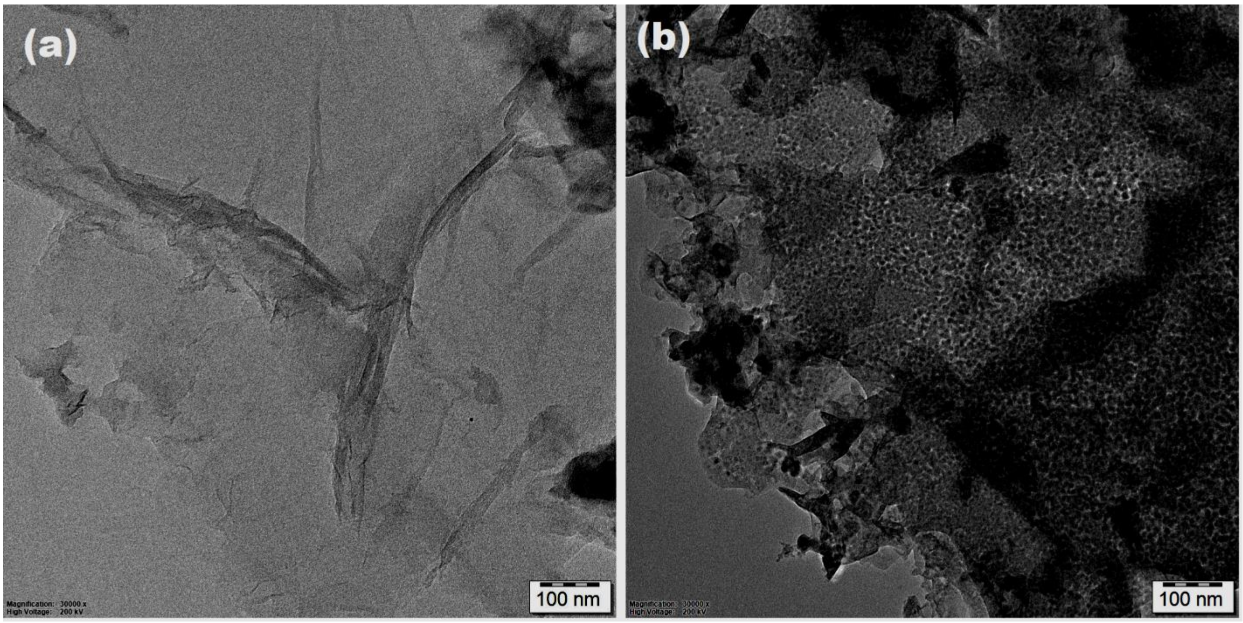

Figure S2. HR-TEM images of (a) rGO, (b) rGO-ZnS

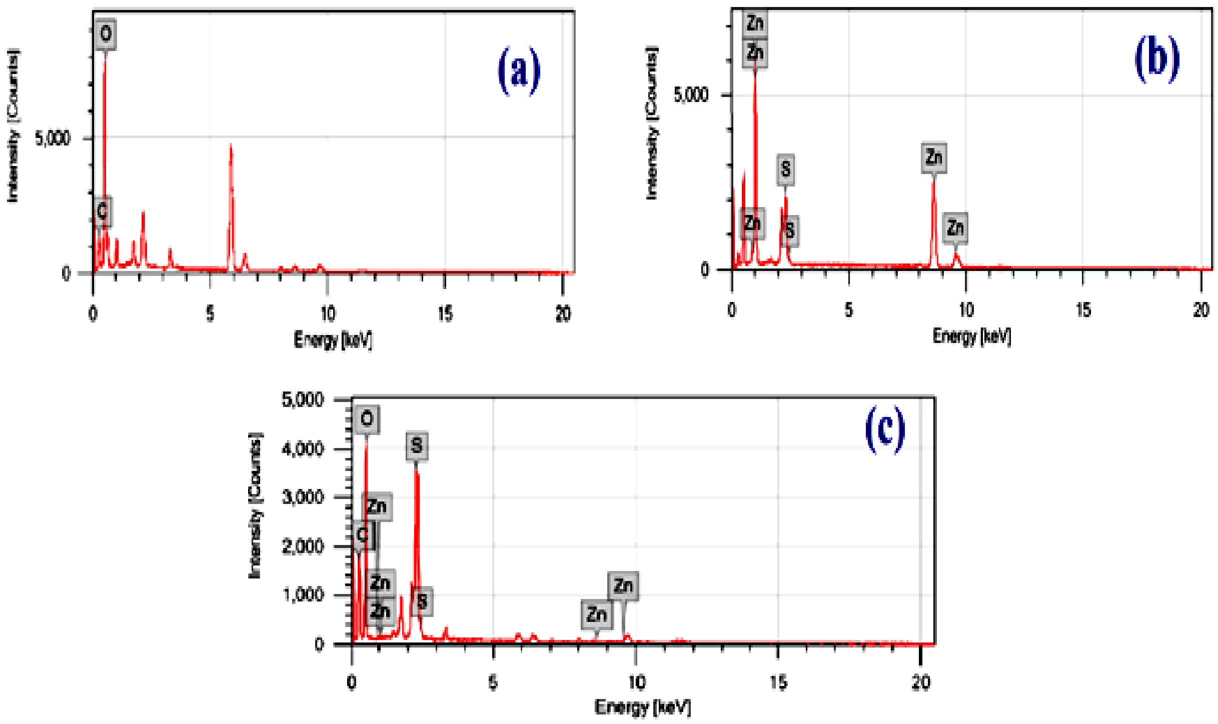

Figure S3. EDX spectra of (a) rGO (b) ZnS (c) rGO-ZnS

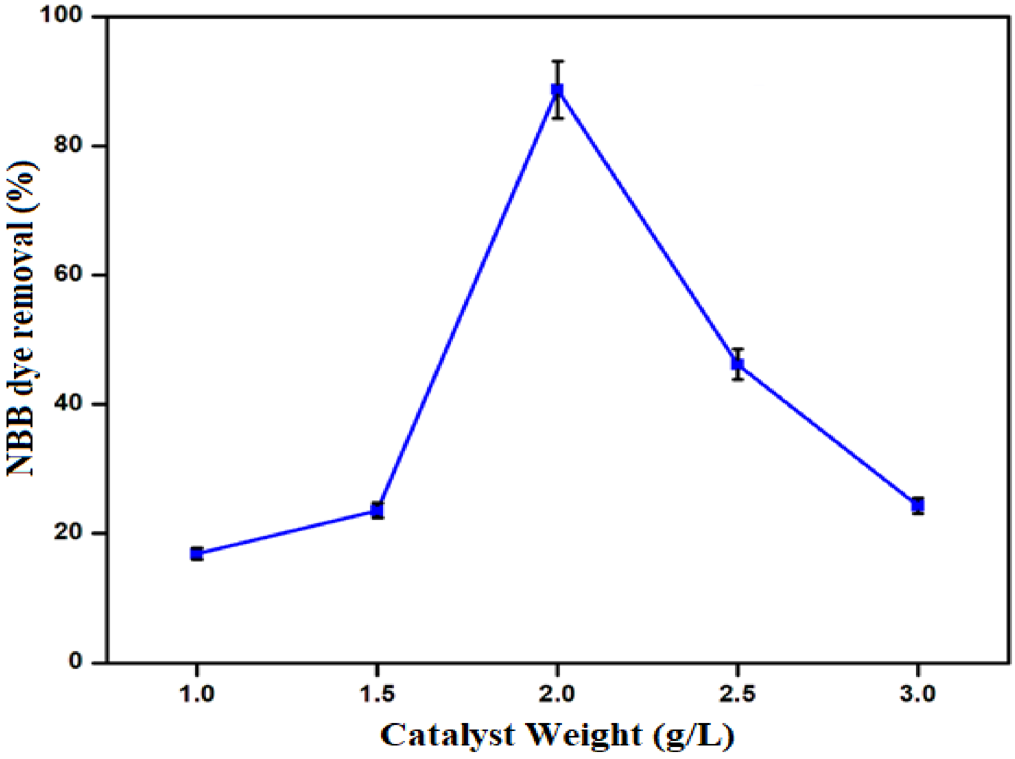

Figure S4. Effect of catalyst weight on the photocatalytic degradation of NBB using solar light: [NBB] = 2×10^{-4} mol/L, pH = 7.0±0.1, airflow rate = 8.1 mL s^{-1}, irradiation time = 90 min, I_{solar} = 1250 × 100 ± 100 lux.

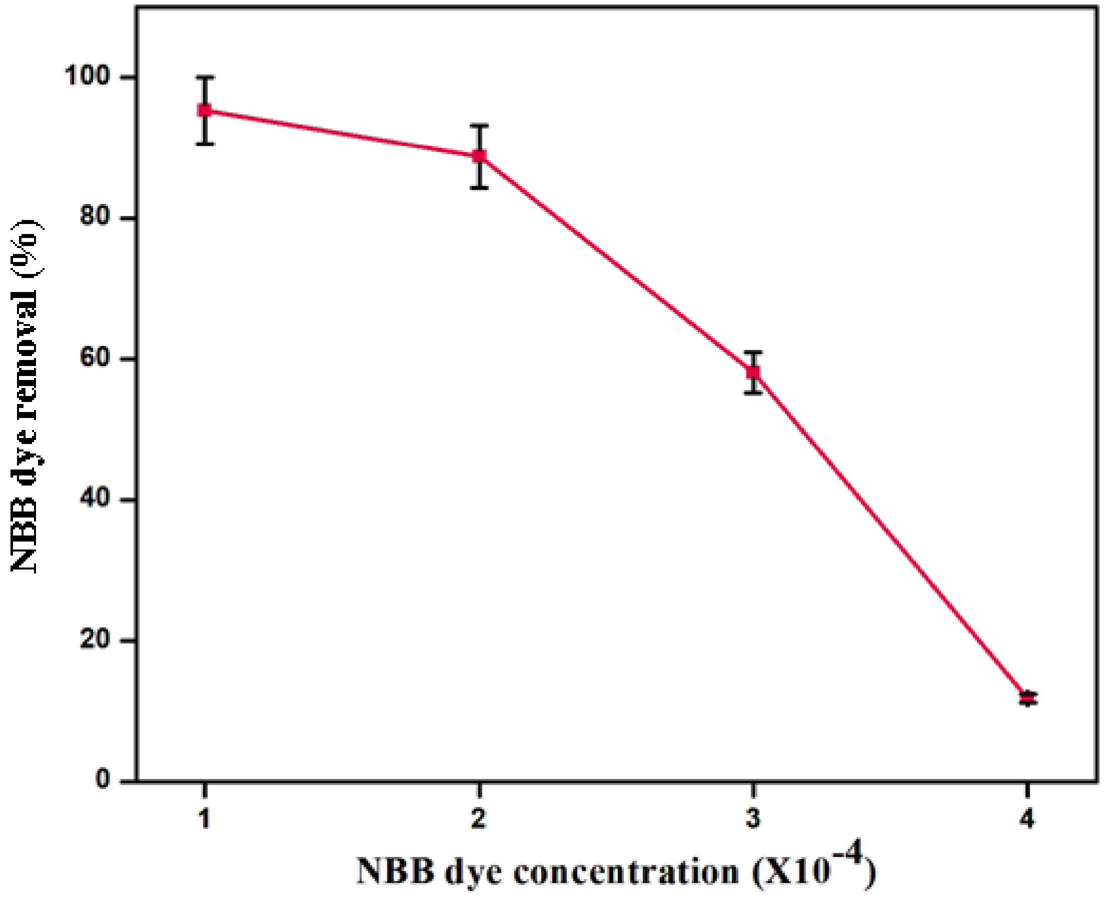

Figure S5. Effect of various initial dye concentrations on the degradation of NBB using solar light/rGO-ZnS nanoparticles: pH = 7.0±0.1, rGO-ZnS nanoparticles = 2g/L, airflow rate = 8.1 mL s^{-1}, irradiation time = 90 min, I_{solar} = 1250 × 100 ± 100 lux.

Table S1. Effect of different wt% of rGO on ZnS for the NBB dye degradation under solar light

Wt% rGO	% of NBB dye degradation
9.3	74.8
11.4	78.5
13.3	88.7
15.2	69.6
17.0	53.7

[NBB] = 2×10^{-4} mol/L, catalyst suspended = 2 g/L, pH = 7, airflow rate = 8.1 mL s^{-1}, irradiation time = 90 min.

The photocatalytic activities of the rGO-ZnS catalysts with 9.3, 11.4, 13.3, 15.2 and 17.0 wt% of rGO loading were evaluated by the degradation of NBB under solar light and the results are given in **Table S1**. As the concentration of rGO is increased from 9.3 to 13.3 wt%, the percentage of degradation increased from 74.8 to 88.7% in NBB degradation. Catalyst loaded with 13.3 wt% of rGO shows a higher percentage of degradation. Further increase of rGO content decreases the percentage of degradation. Hence, 13.3 wt% of rGO is found to be optimum concentration of rGO in ZnS.

Table S2. Rate constants of photocatalytic degradation of NBB dye using solar light/rGO-ZnS nanoparticles

**[NBB] x10^{-4} mol/L**	**k'(min^{-1})**
1	0.0198
2	0.0148
3	0.0101
4	0.0080

Table S3. Comparison of different modified ZnS towards pollutant degradation

**S.No**	**Catalysts**	**Light source**	**Pollutants/ Concentration**	**Catalyst amount**	**%Degradation/ time (min)**	**Ref.**
1	ZnO@ZnS	18 W- UV, 265 nm	CR/40 ppm	50 mg/ 100 mL	89/120	[S1]
2	ZnS@WO_3@ $CoFe_2O_4$	Tungsten halogen lamp (simulated sunlight), > 400 nm	MB/50 ppm	50 mg/-	95.97/120	[S2]
3	CdS/ZnS QDs	Sunlight, 1048 Wm^{-2}	IC/10 ppm	100 mg/ 100 mL	87.2/360	[S3]
4	Ag_2WO_4 decorated ZnS	800 W halogen lamp, visible light	MB/10 ppm	3 mg/ 20 mL	84/140	[S4]
5	ZnS/MoS_2/Bi_2WO_6	500 W Xe lamp	RhB/10 ppm	20 mg/ 50 mL	91.1/120	[S5]
6	rGO-ZnS	Solar light (1250X100±100 lux)	NBB/(2 x 10^{-4}M)*	100 mg/50 mL	93.7/150	Present work

CR- Congo red; MB- Methylene blue; IC- Indigo carmine; RhB- Rhodamine B; NBB- Naphthol blue black; *- concentration given in molar

References

[S1] Sadollahkhani, A.; Nur, O.; Willander, M.; Kazeminezhad, I.; Khranovskyy, V.; Eriksson, M.O.; Yakimova, R.; Holtz, P. O. A detailed optical investigation of ZnO@ZnS core–shell nanoparticles and their photocatalytic activityat different pH values, *Ceram. Int.* **2015**, *41*, 7174–7184

[S2] Palanisamy, G.; Bhuvaneswari, K.; Bharathi, G.; Pazhanivel, T.; Grace, A. N.; Pasha, S.K.K. Construction of magnetically recoverable $ZnS@WO_3@CoFe_2O_4$ nanohybrid enriched photocatalyst for the degradation of MB dye under visible light irradiation, *Chemosphere*, **2021**, *273*, 129687.

[S3] Janbandhu, S.Y.; Suhaila, C.T.; Munishwar, S.R.; Jayaramaiah, J.R.; Gedam, R.S. Borosilicate glasses containing CdS/ZnS QDs: A heterostructured composite with enhanced degradation of IC dye under visible-light, *Chemosphere*, **2022**, *286*, 131672.

[S4] Kokilavani, S.; Al-Farraj, S. A.;. Thomas, A. M., El-Serehy, H. A.; Raju, L. L.; Sudheer Khan, S. Enhanced visible light driven photocatalytic and antibacterial activities of Ag_2WO_4 decorated ZnS nanocomposite, *Ceram. Int.* **2021**, *47*, 12997–13006.

[S5] Liu, D.; Gong, J.; Sun, C.; Xu, L.; Song, Y. Enhanced photocatalytic activity for degradation of ofloxacin and dye by hierarchical flower-like $ZnS/MoS_2/Bi_2WO_6$ heterojunction: Synergetic effect of 2D/2D coupling interface and solid sulfide solutions, *Catal. Commun.* **2022**, *172*, 106546.
